# Transdural Skull Base Infiltration by Glioblastoma: Case Report and Review of the Literature

**DOI:** 10.1155/2023/4727288

**Published:** 2023-01-25

**Authors:** Michael Thrull, Khaled Atasi, Lennart-Maximilian Boese, Mahmut Cakar, Ullrich Heller, Nils Jansen, Leoni-Christine Menzel, Hassan Omaimen, Katharina Theis, Damir Karacic, Diyan Dimov, Roland Coras, Randolf Klingebiel

**Affiliations:** ^1^Department of Neuroradiology, University Hospital OWL, Campus Bielefeld-Bethel, Bielefeld, Germany; ^2^Department of Neurosurgery, University Hospital OWL, Campus Bielefeld-Bethel, Bielefeld, Germany; ^3^Department of Neuropathology, University Hospital Erlangen, Erlangen, Germany

## Abstract

We report the rare occurrence of a temporal glioblastoma multiforme (GBM) showing transdural tumor extension into adjacent mastoid cells. As the dura mater provides a barrier to intraaxial tumors, GBM seldom penetrates into the skull base, even though it is a high-grade astrocytoma with a tendency to spread. Yet, some mechanisms of GBM-induced skull invasion have been identified, making this entity a very rare but nonetheless relevant differential diagnosis in otherwise ambiguous cases of an intracerebral tumor extending into the skull base. In addition, imaging markers that may assist in distinguishing extra- from intraaxial tumor infiltration of the temporal bone are described.

## 1. Introduction

GBM is the most common primary malignant tumor of the central nervous system (CNS). It accounts for approximately 45% of tumors in this category, with an annual incidence of up to 5 per 100,000 persons [1]. As GBM is typically confined to the CNS, no TNM classification for the assessment of cancer spreading has been established.

Although a rare finding, GBM metastases have been reported. Organs affected by extraneural metastases in GBM patients include the pleura and/or lung (60%), lymph nodes (51%), bones (31%), and liver (22%). In those cases with skeletal metastases, the spine (73%), ribs (23%), sternum (18%), skull (14%), and acetabulum (9%) were the most common sites of GBM metastases [2].

The incidence of extraneural metastasis of GBM has been defined as being as low as 0.2% [3]. Primary transdural skull base infiltration by glioblastoma represents an even rarer finding [4].

## 2. Case

**Figure 1:**
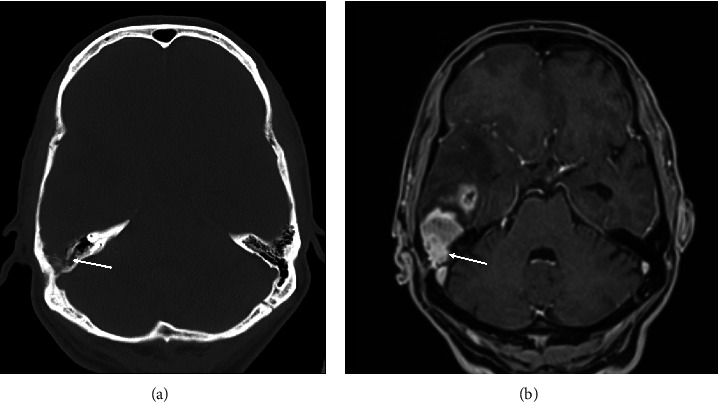
Axial plain CT (a) and contrast-enhanced MRI (b) at the level of the petrous ridges. (a) CT visualizes the osseous destruction of the right petrous ridge (arrow). (b) Contrast-enhanced T1-weighted MRI discloses infiltration of the GBM into the mastoid cells of the right temporal bone (arrow).

**Figure 2:**
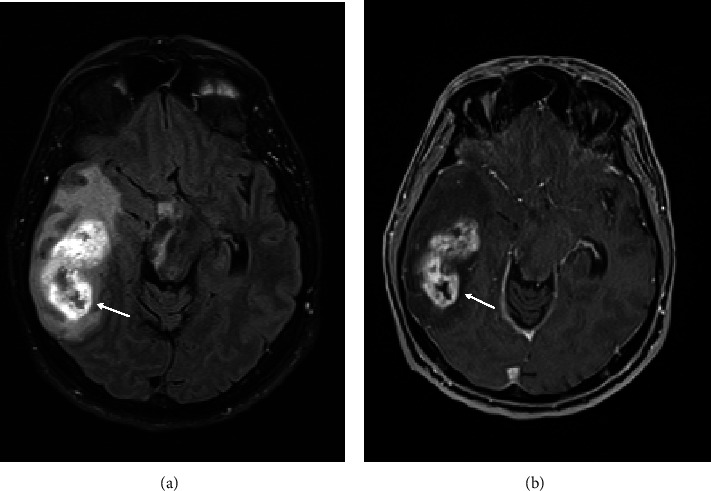
Axial, contrast-enhanced MR images at the middle cranial fossa level. (a) FLAIR and (b) T1-weighted images demonstrate an irregular enhancing mass lesion with central areas of necrosis within the right temporal lobe.

A 61-year-old male patient presented to the emergency department because of left-sided brachiofacial hemiparesis. The patient's history was otherwise unremarkable. The cranial computed tomography (CCT) on admission disclosed a right temporal mass lesion with perifocal edema and lytic changes of the adjacent petrous bone (Figure 1(a)), indicative of tumorous infiltration. Corresponding to the lytic bone changes seen on CCT, there was an extension of contrast-enhancing tumor components into the mastoid cells on contrast-enhanced magnetic resonance imaging (MRI; Figure 1(b)). MRI otherwise revealed an enhancing, cystic necrotic tumor, showing marked perifocal edema within the right temporal lobe (Figures 2(a) and 2(b)). Audiometric testing disclosed a hearing impairment.

Tumor resection was subsequently performed via temporal craniotomy. Neuromonitoring and 5-aminolevulinic-acid (5-ALA)-fluorescence were used during surgery.

Intraoperatively, glioblastoma-like tissue with necrotic areas, thrombosed vessels, and an infiltration zone at the tumor margins was encountered. Following macroscopically complete resection, re-resection was performed under 5-ALA fluorescence, resulting in a 5-ALA-negative tumor resection cavity.

Early postoperative MRI confirmed complete intracranial resection, while questionable residual tumor was disclosed in the deep right mastoid cells.

**Figure 3:**
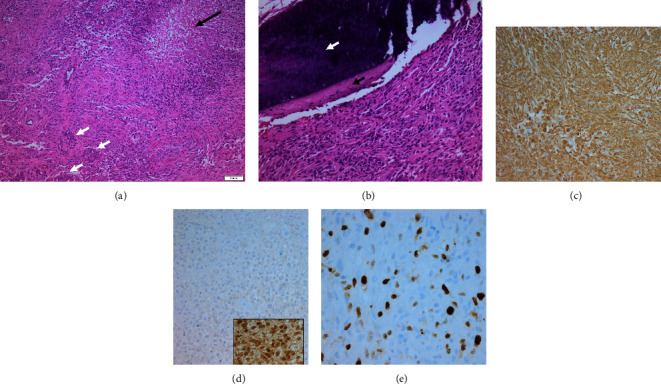
(a) HE staining showing a malignant glial tumor with histomorphological features of a glioblastoma, IDH wild-type (CNS WHO Grade 4). Black arrow: palisading necrosis. White arrows: microvascular proliferation. (b) The tumor shows areas with close association to fragments of the dura (black arrow) and bone (white arrow) (HE staining). (c) GFAP immunohistochemistry confirms the glial tumor cells. (d) No IDH1 mutation (R132H); inset in (d) on slide positive control. (e) Ki67 immunostaining highlights increased proliferation activity.

Histopathology, as shown in Figures 3(a)–3(e), revealed Isocitrate dehydrogenase 1 (IDH1) wild-type GBM with methylguanine-DNA methyltransferase (MGMT) promoter methylation.

Adjuvant treatment according to the CeTeG protocol was started including standard radiotherapy with 60 Gy (30 × 2 Gy) and six 42-day cycles with oral administration of Lomustine (CCNU) on days 1 and temozolomide (TMZ) on day 2–6 [5]. The adjuvant treatment was supplemented by tumor treatment fields (TTF, Optune).

## 3. Discussion

Our case showed typical features of a glioblastoma: an irregularly circumscribed, contrast-enhancing tumor with central necrosis, extensive vasogenic edema, and evidence of peritumoral neovascularization.

In 2009, Kwak and Shatzkes [6] cited altogether twelve case reports of transdural tumor infiltration from 1962 to 1996. Since then, only a few additional cases have been documented [4, 7–9].

Extracranial GBM dissemination is explicable in the case of prior skull base injuries, such as craniotomies, biopsies, or shunt placement [6]. Possible routes of transdural infiltration, not facilitated by prior dural defects, include spreading through a dural slit, transdural herniation due to increased intracranial pressure, direct dural infiltration by tumor cells and tumor migration through the dura mater via the cranial nerve foramina.

In our case, no previous skull base surgery was present, making iatrogenic dural defects as the route of infiltration less probable. Yet, the mastoid tegmen is known as a thin bony plate, which may develop defects for various reasons, one of which being tumorous lesions and increased intracranial pressure [10].

In our case, we hypothesize that the combination of a rapidly growing tumor within the medial cranial fossa in combination with the low-resistance mastoid tegmen may have facilitated herniation and subsequent infiltration of tumorous tissue into the upper mastoid cells.

Regarding imaging-based tumor assessment, the absence of a peritumoral cerebrospinal fluid (CSF) boundary (the CSF cleft sign) and a relatively narrow dural attachment (absence of the dural tail sign) helped to differentiate the GBM from a common extraaxial mass such as a meningioma.

## 4. Conclusion

Our case illustrates that in the setting of an ambiguous intracerebral tumor with transdural skull base invasion, glioblastoma represents a rare but nonetheless relevant differential diagnosis.

## Data Availability

The image data used to support the findings of this study are included within the article.

## Abbreviations

GBM: Glioblastoma multiforme
CNS: Central nervous system
CCT: Cranial computed tomography
MRI: Magnetic resonance imaging
5-ALA: 5-aminolevulinic-acid
IDH1: Isocitrate dehydrogenase 1
MGMT: Methylguanine-DNA methyltransferase
CCNU: Lomustine
TMZ: Temozolomide
TTF: Tumor treatment fields
CSF: Cerebrospinal fluid
HE: Hematoxylin and eosin
GFAP: Glial fibrillary acidic protein.
